# Exploring Concomitant Ophthalmic Comorbidities in Portuguese Patients with Inherited Retinal Diseases: A Comprehensive Clinical Study

**DOI:** 10.3390/genes16070743

**Published:** 2025-06-26

**Authors:** Rita Mesquita, Ana Marta, Pedro Marques-Couto, José Costa, Sérgio Estrela-Silva, Diogo Cabral, João Pedro Marques, Sara Vaz-Pereira

**Affiliations:** 1 Department of Ophthalmology, Faculdade de Medicina, Universidade de Lisboa, 1649-028 Lisbon, Portugal; r.mesquita@edu.ulisboa.pt; 2 Department of Ophthalmology, Unidade Local de Saúde de Santo António, EPE (ULSSA), 4099-001 Porto, Portugal; 3 Instituto Ciências Biomédicas Abel Salazar (ICBAS), 4050-313 Porto, Portugal; 4 Department of Ophthalmology, Unidade Local de Saúde de São João, EPE (ULSSJ), 4200-319 Porto, Portugal; 5 Department of Ophthalmology, Unidade Local de Saúde de Braga (ULSB), 4710-243 Braga, Portugal; 6 Department of Ophthalmology, Unidade Local de Saúde de Almada-Seixal, EPE (ULSAS), 2850-267 Lisbon, Portugal; 7 Clinical Academic Centre of Coimbra (CACC), 3000-370 Coimbra, Portugal; marquesjoaopedro@gmail.com; 8 Department of Ophthalmology, Hospitais da Universidade de Coimbra, Unidade Local de Saúde de Coimbra, EPE (ULSC), 3004-561 Coimbra, Portugal; 9 University Clinic of Ophthalmology, Faculty of Medicine, University of Coimbra (FMUC), 3000-370 Coimbra, Portugal; 10 Department of Ophthalmology, Unidade Local de Saúde de Santa Maria (ULSSM), 1649-028 Lisbon, Portugal

**Keywords:** cataract, cystoid macular edema, inherited retinal dystrophies, ophthalmology, retinitis pigmentosa

## Abstract

**Background/Objectives**: Inherited retinal diseases (IRDs) are a heterogeneous group of rare eye disorders characterized by progressive photoreceptor degeneration, leading to severe visual impairment or even blindness. This study aims to investigate the prevalence, types, and clinical significance of ophthalmic comorbidities in Portuguese patients with IRDs. **Methods**: This nationwide Portuguese population-based retrospective study was based on the IRD-PT registry (retina.com.pt). Statistical analysis was conducted using Microsoft^®^ Excel^®^ for Microsoft 365 and IBM SPSS Statistics version 29.0.2.0. Informed consent was obtained from all participants. **Results**: A total of 1531 patients (1254 families) from six centers were enrolled. The cohort consisted of 51% males, with a mean age of 45.8 ± 19.3 years and a mean age at diagnosis of 39.4 ± 19.5 years. Overall, ocular comorbidities were reported in 644 patients (42.1%). In 176 individuals (11.5%), multiple concurrent comorbidities were found. Cataract was the most common comorbidity (21.3%), followed by amblyopia (6.3%) and high myopia (5.9%). Statistically significant associations with ocular comorbidities were observed in isolated progressive IRDs. Specifically, AR RP was associated with cataract (*p* < 0.001), and gene analysis revealed several significant associations. CRB1 was statistically linked to epiretinal membrane (ERM) (*p* = 0.003), EYS with cataract (*p* = 0.001), PROM1 with choroidal neovascularization (CNV) (*p* = 0.0026), and USH2A with macular hole (*p* = 0.01). Patients with the RPE65 mutation in Leber congenital amaurosis were associated with ERM (*p* = 0.019). There was also a significant association between X-linked RP and high myopia (*p* < 0.001) and CNV in Best disease (*p* < 0.001); in syndromic IRDs, cataract, cystoid macular edema, and ERM were observed in Usher syndrome, *p* = 0.002, *p* = 0.002, and *p* = 0.005, respectively, and the MYO7A gene was linked to cataract (*p* = 0.041) and strabismus (*p* = 0.013); pseudoxanthoma elasticum was significantly associated with CNV (*p* = 0.002); and foveal hypoplasia was associated with anterior segment dysgenesis (*p* < 0.001). **Conclusions**: This study enhances the current understanding of ocular comorbidities in IRDs in Portuguese patients. Common findings were cataract, refractive error, and CME. Stationary IRDs and pattern dystrophies showed fewer concomitant comorbidities, supporting their classification as non-progressive or benign conditions. The significance of registries like IRD-PT cannot be overstated, particularly in the context of rare diseases. These databases serve multiple crucial functions in enabling detailed documentation of disease characteristics and long-term monitoring of disease progression.

## 1. Introduction

Inherited retinal diseases (IRDs) are a group of phenotypically and genetically heterogenous rare eye diseases primarily caused by the degeneration or apoptosis of photoreceptors [1]. Over 345 different disease-causing genes have been identified in association with IRDs. Overall, the cumulative prevalence of IRDs is 1:3450 individuals [2]. Retinitis Pigmentosa (RP) is the most common IRD, with a prevalence of approximately 1:4000 worldwide [3,4,5]. These diseases can be present at birth, manifest in early childhood, or appear even later in life, and their inheritance pattern can be autosomal dominant (AD), recessive (AR), X-linked (XL), digenic, or mitochondrial [1,6]. Due to the progressive nature of most IRDs, a significant impact can be seen on the quality of life and life choices of those affected. Since curative treatment is seldom available, understanding the prevalence of treatable ophthalmic comorbidities in this population group is extremely important to provide better care to IRD patients [7].

There are different classifications of IRDs. The IRD-PT (retina.com.pt) registry divides IRDs into seven main groups: isolated progressive IRDs, isolated stationary IRDs, syndromic IRDs, inner retina and/or vitreoretinal dystrophies, chorioretinal dystrophies, inherited optic neuropathies, and other rare disorders of the posterior segment of the eye [8,9]. The importance of registries such as the IRD-PT is particularly pronounced in the context of rare diseases, where comprehensive data collection is essential for advancing research and improving patient outcomes.

In several studies, IRDs have been associated with ocular comorbidities such as cataract, cystoid macular edema (CME), choroidal neovascularization (CNV), and refractive errors (Table 1).

Woon et al. suggested that IRD patients have a higher incidence of cataract in younger patients and that older patients are more susceptible to the development of CME [4]. In addition to the two conditions described above, a study by Iuliano et al. also described the presence of epiretinal membrane (ERM) [16]. Studies like these are important since they illustrate the need to recognize causes of treatable visual loss, such as cataract, CNV, and refractive error, in these patients to provide improved care and a better quality of life [17,18]. The same study by Woon et al. found that in Taiwan the prevalence of cataract, CME, ERM, RD, and retinoschisis in 403 IRD patients was 8.2%, 6.5%, 0.5%, 0.5%, and 0.3%, respectively. When compared with a population without IRDs, IRD patients had a higher incidence of cataract (8.2% vs. 1.5%) and CME (6.5% vs. 0.5%).

This study aims to investigate the prevalence, types, and clinical significance of ophthalmic comorbidities in a Portuguese cohort of IRD patients.

## 2. Materials and Methods

This was a nationwide Portuguese population-based retrospective study using the IRD-PT registry (retina.com.pt) [8,9]. In the classification used in the IRD-PT registry, IRDs are coded according to the International Classification of Diseases (ICD) 9, ICD 10, ICD 11, and Orphanet Rare Disease Ontology (ORPHA) codes [19]. This registry is mainly used for retinal research, and patient identification is encrypted. The IRD-PT informed consent was approved by the Portuguese General Data Protection Regulation and the Local Ethics Committee. All patients signed the IRD-PT informed consent, and the study was conducted following the tenets of the Declaration of Helsinki [20].

### 2.1. Clinical and Demographical Data

Clinical and demographic data, including age, gender, district of residence, hospital of follow-up, consanguinity, family history, age of symptom onset, age at diagnosis, visual acuity, symptoms, previous treatments, type of IRD, and identified genes were extracted from the IRD-PT registry. Additional diagnoses coded in the registry with human phenotype ontology terms included the following: high hyperopia, high myopia, amblyopia, lamellar hole, macular pseudohole, macular hole, cataract, retinal detachment (RD), anterior segment dysgenesis (ASD), CME, strabismus, glaucoma, ERM, CNV, keratoconus, and vitreomacular traction.

### 2.2. Statistical Analysis

For the statistical analysis, Microsoft^®^ Excel^®^ for Microsoft 365 MSO (Version 2410 Build 16.0.18129.20158) 64-bit and IBM SPSS Statistics version 29.0.2.0 were used. Statistical analysis was performed using both the chi-square test of independence and Fisher’s exact test, depending on the expected frequencies in each category. For categorical variables where at least 80% of the outcomes had expected frequencies ≥ 5, the chi-square test of independence was employed. In cases where this criterion was not met, Fisher’s exact test was utilized instead. The statistical significance level was set at *p* ≤ 0.05. These analyses were conducted to compare the prevalence of various ophthalmological conditions between groups. For each comparison, contingency tables were created, and appropriate test statistics and *p*-values were calculated. The results of these analyses were used to identify significant associations between a specific disease and the presence of specific ophthalmological conditions as well as between specific genes and comorbidities.

## 3. Results

This study included 1531 patients with a clinical diagnosis of IRD from six different hospitals in Portugal: Unidade Local de Saúde (ULS) de Coimbra, ULS de Santo António, ULS de Santa Maria, ULS de Braga, ULS de São João, and ULS de Almada-Seixal.

### 3.1. Clinical and Demographical Characteristics

The clinical and demographic characteristics of the 1531 patients (1254 families) are outlined in Table 2.

**Figure 1:**
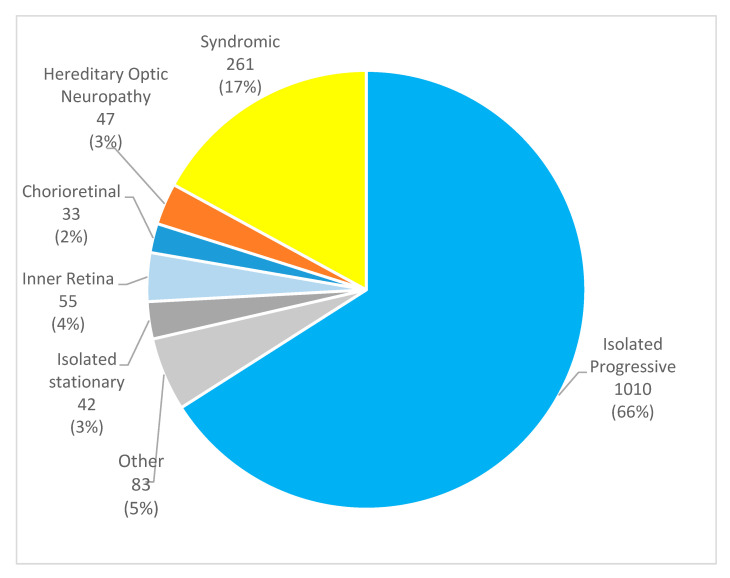
Pie chart demonstrating the distribution of the different disease subgroups in the study sample, *n* (%).

The most frequent subgroup was isolated progressive retinal disorders, which represented 66% of the sample (1010 patients). Figure 1 illustrates the distribution of the sample.

Regarding ocular comorbidities, we found that most patients did not present any complications and that the most frequent ones were cataract, followed by amblyopia, high myopia, and glaucoma. Table 3 details the frequency of ocular comorbidities in the sample.

In the analysis of associated diagnoses, we found that 644 (42.1%) of patients presented ophthalmic comorbidities. While 446 (29.1%) patients presented with isolated conditions, 198 (12.9%) patients had multiple concurrent conditions. Among the patients with multiple diagnoses, notable combinations included cataract with CME (*n* = 27, 1.8%), cataract with ERM (*n* = 22, 1.43%), cataract with glaucoma (*n* = 14, 0.9%) and cataract with high myopia (*n* = 13, 0.9%). Our data also revealed that some patients presented with three concurrent diagnoses, albeit in smaller numbers—for instance, ERM, CME, and cataract were found together in 8 patients (0.5%).

### 3.2. Isolated Progressive Retinal Disorders

This was the largest subgroup with 1010 patients, representing 66.0% of the sample. For a better analysis, the data was separated into three subgroups, namely, peripheral, macular, and pattern dystrophies with 785 (77.7%), 191 (18.9%), and 34 (3.7%) patients, respectively.

The peripheral dystrophies group included 785 patients, with RP being the most prevalent disease, totaling 605 patients.

The prevalence of ocular comorbidities varied across the IRD subtypes. Cataract was the most frequent comorbidity, observed across multiple IRD groups. Overall, RP had a prevalence of cataract of 36.4%, and there was a statistical association between this comorbidity and AR RP (*p* < 0.001). In patients with XL RP, we found a statistical association with high myopia, which was also the most prevalent comorbidity in this group (25.9%, *p* < 0.001). Other notable comorbidities and their associated IRDs were as follows: CME in AR RP (12.9%, *p* < 0.001); ERM in AR RP (12.2%, *p* < 0.001); and strabismus in LCA (14.0%, *p* < 0.001). Additionally, LCA presented with keratoconus (*p* < 0.001).

**Figure 2:**
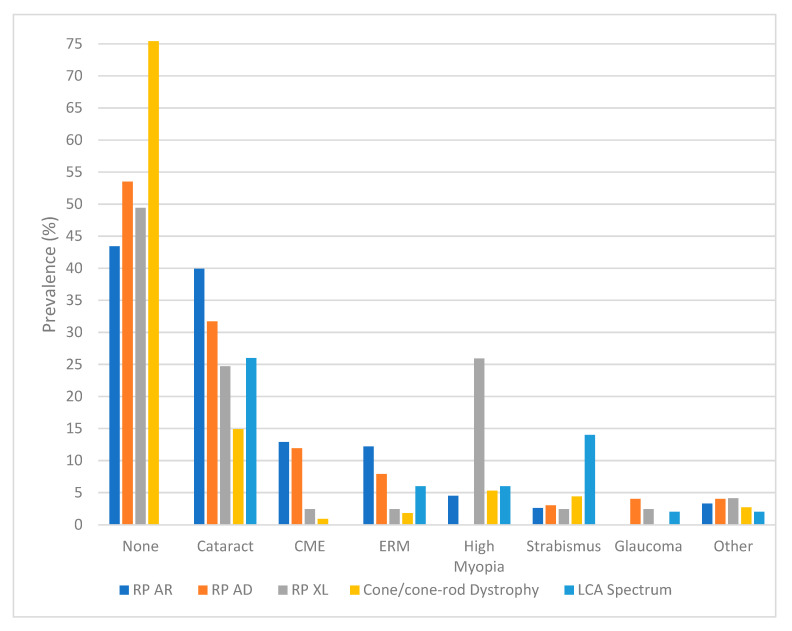
Bar graph detailing the different comorbidities present in the peripheral isolated IRDs. The “Other” category includes amblyopia, high hyperopia, lamellar hole, macular hole, vitreomacular traction, retinal detachment, keratoconus, choroidal neovascularization, and pseudohole. AR: autosomal recessive; AD: autosomal dominant; CME: cystoid macular edema; ERM: epiretinal membrane; LCA: Leber congenital amaurosis; XL: X-linked.

When comparing the prevalence of specific genes and comorbidities, we found the following associations in AR RP: CRB1 was statistically associated with ERM (*p* = 0.003), EYS with cataract (*p* = 0.001), PROM1 with CNV (*p* = 0.0026), and USH2A with macular hole (*p* = 0.01). Regarding LCA, there was a statistical association between ERM and RPE65 (*p* = 0.019). Figure 2 further details the different comorbidities recorded in this study.

Regarding comorbidities within the cohort of patients with macular dystrophies, there were a total of 192 (19.0%) patients. The most frequent pathology was Stargardt disease (STGD), with the most frequent comorbidity being cataract. Patients with STGD were associated with complication-free disease (*p* < 0.001). The most frequent complications in Best disease (BD) were cataract and CNV. There was a statistical association between CNV and BD (*p* < 0.001). Table 4 further details the comorbidities in this group.

Pattern dystrophies comprised 34 (2.2%) subjects in the cohort of isolated progressive IRDs. Adult-onset vitelliform pattern dystrophy represented the predominant subtype (*n* = 17, 50.0%), followed by multifocal pattern dystrophy (*n* = 7, 20.6%) and butterfly-shaped pattern dystrophy (*n* = 5, 14.7%). In the adult-onset vitelliform pattern dystrophy group, most patients (70.6%) had no ocular comorbidities. Cataract was the most frequently observed comorbidity (17.6%). Among patients with multifocal pattern dystrophy, 42.9% developed cataract, while 57.1% remained comorbidity-free. In the butterfly-shaped pattern dystrophy group, 40% of patients presented with cataract. Notably, all patients with reticular pattern dystrophy had cataract. Figure S1 of the Supplementary File S1 illustrates the different comorbidities found in this group.

### 3.3. Isolated Stationary Retinal Disorders

The study population comprised 42 subjects, distributed as follows: 21 subjects (50%) diagnosed with achromatopsia, 11 subjects (26.2%) with Congenital Stationary Night Blindness (CSNB), and 10 subjects (23.8%) with fundus albipunctatus. Regarding ocular comorbidities, most patients remained comorbidity-free. All patients with fundus albipunctatus (100%) presented without concurrent ophthalmic conditions. In the achromatopsia cohort, the majority (85.7%) were comorbidity-free, with cataract observed in 9.5% of cases. Among CSNB patients, 72.7% had no ocular comorbidities, while high myopia was the most frequently associated condition (18.2%). An overview of the ocular comorbidities observed in this cohort is presented in Figure S2 from the Supplementary File S1.

### 3.4. Syndromic Retinal Disorders

This group (*n* = 261, 17.0%) had multiple different syndromes, with the most common being Usher syndrome, representing 38.3% of the sample. This group had similar results to the isolated AR RP group, with cataract being the most common comorbidity followed by CME. Through this study, we found an association between ocular comorbidities and Usher syndrome, namely, cataract (*p* = 0.002), CME (*p* = 0.002), and ERM (*p* = 0.005). Analysis also revealed a statistically significant association between MYO7A mutations and both cataract (*p* = 0.041) and strabismus (*p* = 0.013).

Bardet–Biedl Syndrome (BBS) was the second most frequent syndrome and had a similar rate of comorbidity-free patients (40.5%) and cataract. The third most prevalent disease in this sample was pseudoxanthoma elasticum (PXE), where a significant proportion had CNV. There was a statistical association between PXE and CNV (*p* = 0.002). Even though the sample size was small, patients with Knobloch syndrome seemed to present with more comorbidities than the remaining, with only one (16.7%) patient out of six being comorbidity-free. Table 5 further details the comorbidities present in this group.

### 3.5. Inner Retina and/or Vitreoretinal Dystrophies

There were 55 (3.6%) patients in this group. The most prevalent disease was XL retinoschisis with 28 (20.9%) patients. Most patients did not have ophthalmic comorbidities, with cataract being the most frequently reported condition. Among those with Wagner syndrome (18.2%), the majority were comorbidity-free. In the Stickler syndrome subgroup (16.4%), over half of the patients had no additional ocular conditions, while the most reported comorbidities included high myopia and RD. All five patients (9.1%) diagnosed with Goldmann–Favre Syndrome were free of ocular comorbidities.

Figure S3 (Supplementary File S1) illustrates the prevalence and distribution of ocular comorbidities across the different disease subtypes within this group.

### 3.6. Chorioretinal Disorders

A total of 33 patients (2.2% of the total cohort) were diagnosed with chorioretinal disorders. Choroideremia was the most common condition, affecting 11 patients (33.3%), of whom 63.6% were comorbidity-free. Cataract was the most frequently observed comorbidity in this subgroup. Central areolar choroidal dystrophy was the second most frequent diagnosis (27.3%), with a significant proportion of patients presenting without comorbidities. Among patients with Bietti crystalline dystrophy (21.2%), 57.1% were comorbidity-free, with ERM being the most common comorbidity (28.6%). Gyrate atrophy accounted for 15.2% of cases, with 40.0% of patients showing no ocular comorbidities.

Figure S4 (Supplementary File S1) provides a detailed overview of the comorbidities associated with these chorioretinal dystrophies.

### 3.7. Hereditary Optic Neuropathy

There was a total of 47 patients in this subgroup. The most common disease was AD optic atrophy with 33 patients (70.2%), of whom 93.9% were comorbidity-free. Glaucoma was the most frequent comorbidity, reported in 6.1% of patients. Among patients with Leber hereditary optic neuropathy (12.8%), the majority (66.7%) had no ocular comorbidities. All patients (17.0%) diagnosed with other hereditary optic neuropathies had no documented ocular comorbidities. Figure S5 (Supplementary File S1) illustrates the distribution of ocular comorbidities across these hereditary optic neuropathy subtypes.

### 3.8. Other

This category included 83 patients, with foveal hypoplasia being the most prevalent diagnosis (31.3%). Among these, 38.5% were comorbidity-free. The most frequent ocular comorbidities in this group were cataract and strabismus, each observed in 26.9% of cases. Notably, 23.9% of patients with foveal hypoplasia had ASD, and the statistical analysis confirmed a significant association between ASD and foveal hypoplasia (*p* < 0.001). Glaucoma was present in 15.4% of cases, while high hyperopia was seen in 7.7%.

The second most common diagnosis was oculocutaneous albinism (26.5%), with 72.7% of patients presenting without ocular comorbidities. Ocular albinism was less frequent, and most of these patients were also comorbidity-free.

Other less common conditions included coloboma (6.0%), microphthalmia–retinitis pigmentosa–foveoschisis–optic disc drusen syndrome (4.8%), and extensive macular atrophy with pseudodrusen-like appearance (4.8%), with no consistent comorbidity patterns beyond small clusters of cataract and strabismus. Among patients with unspecified inherited retinal diseases (19.3%), 93.8% had no associated comorbidities. Figure S6 (Supplementary File S1) further details the comorbidities present in this subgroup.

## 4. Discussion

In this study we investigated the prevalence, types, and clinical significance of ophthalmic comorbidities in a Portuguese IRD cohort.

The present study can be compared to recent literature regarding ocular comorbidities in patients with IRDs. Several studies have highlighted the importance of recognizing treatable causes of visual loss in IRD patients, such as cataract, CNV, and refractive errors, to provide improved care and better quality of life [17,18]. Our findings support this approach, showing that a significant proportion of patients (42.1%) presented with potentially treatable ocular comorbidities.

A study conducted by Woon and colleagues in Taiwan revealed that among 403 IRD patients, the prevalence of ocular comorbidities varied significantly. They documented the presence of cataract in 8.2%, CME in 6.5%, ERM in 0.5%, and RD in 0.5% of the sample [4]. Our study found higher rates of cataract (21.2%) and ERM (2.0%), but similar rates of CME (4.1%) and RD (0.8%). The difference between the prevalence of cataract may be influenced by external factors such as smoking and UV-B exposure, as well as genetic factors, since the average age of the samples is quite similar (49.2 ± 18.6 vs. 45.8 ± 19.3 years) [21]. While data on the smoking habits of the patients was not collected, the prevalence of smoking in Portugal is higher than in Taiwan, with 21% of adults smoking in Portugal in comparison to 13.1% of adults in Taiwan [22,23].

Multiple studies have investigated the prevalence of these different comorbidities in patients, especially the ones with typical RP. Tan et al. reported a cataract prevalence of 44% in a Chinese cohort, while Iuliano et al. found 72.5%, and Testa et al. noted 52.2%—figures that align closely with the present results [16,24,25]. This consistency in the prevalence of cataract may stem from cytokines released by degenerated retinal tissue, which could impair the blood–ocular barrier, or from oxidative stress, as hypothesized in prior research [26].

Regarding macular comorbidities, previous studies show varying prevalence rates. Testa et al. reported CME in 20% and ERM in 16% of patients, while Tan et al. found ERM in 59.4% and CME in 21.4%, and Marques et al. noted CME in 24.5% and ERM in 64.5% [27]. The prevalence of these conditions is higher than what was found in our study. Notably, XL RP patients appear to exhibit fewer macular comorbidities than other inheritance types. Additionally, our findings are consistent with the 2.0% glaucoma prevalence reported by Tan et al. [16,24,25].

In this study, we identified a statistically significant association between CRB1 mutations and the presence of ERM, a finding that diverges notably from previous reports which consistently emphasized CME as the predominant macular pathology in CRB1-associated retinopathies [27,28]. However, a case report of two patients also found the presence of an ERM [29]. Our findings therefore challenge the current paradigm and raise important questions about the phenotypic variability of CRB1-associated retinopathies.

Our genetic analysis revealed a statistically significant association between EYS mutations in AR RP and the presence of cataract, compared to patients with mutations in other AR RP-associated genes. This finding suggests that EYS-related RP may predispose individuals to an increased risk of lens opacities or earlier cataract development. This association is supported by findings from a study by Silva Martins et al., which reported that 55% of patients in a Brazilian cohort with EYS-related RP either had cataract or had undergone cataract surgery [30]. Their results align with our data and underscore the clinical importance of monitoring cataract development in this specific genetic subgroup of RP patients.

The present study also identified a statistically significant association between PROM1-related RP and the presence of CNV, suggesting that CNV may be a more frequent complication in this genotype than previously recognized. This finding contrasts with the current clinical literature, which more commonly describes macular atrophy as the primary manifestation of central retinal involvement in PROM1-associated retinal dystrophies. For example, Fujinami et al. emphasized that PROM1 mutations are typically associated with macular dystrophy or atrophy rather than neovascular complications [31]. However, our results are supported by mechanistic insights from experimental models. In particular, Bhattacharya et al. demonstrated that PROM1-null Xenopus develop early retinal pigment epithelium dysfunction and subretinal drusenoid-like deposits resembling those seen in age-related macular degeneration, a condition frequently complicated by CNV [32]. These deposits may disrupt the retinal pigment epithelium–Bruch’s membrane–choriocapillaris interface, creating a proangiogenic microenvironment that could facilitate CNV formation through chronic oxidative stress and VEGF upregulation. Taken together, these findings underscore the biological plausibility of CNV in PROM1-RP and highlight the need for larger, genotype-specific studies to further clarify the prevalence, risk factors, and clinical course of CNV in this population.

Overall, our study highlights both alignment and divergence with existing literature. While cataract prevalence mirrors previous reports, especially by Tan et al., the variability in macular comorbidities underscores the heterogeneity of RP across populations and inheritance patterns. Consistently reported glaucoma rates reinforce its importance as a relevant, albeit secondary, comorbidity in RP.

The statistical association between XL RP and high myopia corroborates findings by Sieving et al. [33]. A more recent study by Lee et al. reported a 16.1% prevalence of high myopia in a Korean cohort of patients with RP, with no statistically significant association observed between refractive error and the pattern of inheritance. In contrast, our study identified a lower overall prevalence of high myopia, at 6.6%, with a statistically significant association in cases of X-linked RP. This suggests the possibility of genetic or environmental influences, since the prevalence of myopia was reported to be higher in Asian populations compared to Western populations [34]. These findings underscore the importance of continued large-scale multicenter studies to better understand the underlying mechanisms driving these variations, and to refine clinical management strategies tailored to specific RP subtypes.

Testa et al. reported a cataract prevalence of 52.2% in individuals with Usher syndrome, whereas our study found a slightly lower prevalence (43.0%). Similarly, the prevalence of CME and ERM was comparable between the studies [35]. As for BBS, another syndromic RP, the study by Castro-Sanchez found a prevalence of cataract of around 50%, which corroborates our results [36].

Our findings demonstrate a statistically significant association between cataract development and MYO7A mutations in patients with Usher syndrome type I, aligning with and extending observations from previous studies. In the multicenter prospective cohort by Testa et al., 37% of patients had cataract and an additional 23.5% were pseudophakic, underscoring the high overall burden of lens pathology in this population [37]. Similarly, Romo-Aguas et al. reported that 60% of MYO7A patients developed cataract, further supporting its relevance as a common and potentially progressive feature of the disease [38]. This suggests that cataract may not only be age-related or secondary to retinal degeneration but may also be influenced by the molecular mechanisms associated with MYO7A dysfunction.

Relative to PXE, this study found that 48.4% of patients had CNV, which is in accordance with the results presented by Bartstra et al., showcasing a prevalence between 44 and 62% depending on the genotype, thus emphasizing the influence of genetic factors in determining CNV risk [39]. This shows the need to better understand the physiopathology of this condition and the relevance of screening and follow-up protocols for these patients.

In patients with STGD, the present study suggests these patients are relatively absent from ocular comorbidities. Curiously, there were no documented cases of CNV, suggesting it is a rare comorbidity. A study by Armstrong et al. with 52 patients only found this comorbidity in 0.06% of patients [40].

Among patients with BD, our study found a cataract prevalence of 17.9%, consistent with rates observed in the general population, suggesting no increased risk in this subgroup [41]. In contrast, Han et al. reported a CNV prevalence of 59.3%, whereas our findings indicate a markedly lower rate [42]. These findings highlight the need for further research to better understand the variability in ocular manifestations among individuals with BD across different populations.

Regarding pattern dystrophies, the limited sample size precludes definitive conclusions. However, adult-onset vitelliform pattern dystrophy—the most prevalent condition within this group—demonstrated a cataract prevalence comparable to that of the general population, further supporting the relatively benign clinical course of these disorders [43]. Similarly, stationary IRDs seemed to have a relatively benign course since most patients did not present comorbidities, further corroborating the idea that these are isolated and non-evolutive diseases.

In this study, the prevalence of cataract among patients with XL retinoschisis was 21.7%, aligning with rates observed in the general population. This suggests that individuals with XL retinoschisis may not be at increased risk for cataract development. However, a study by Zhang et al. reported a CNV prevalence of 8.1% among patients, whereas no cases of CNV were identified in our study cohort [44]. Additionally, it has been reported that around 20% of patients develop RD, which differs from the present results with only 7.1% of patients exhibiting this comorbidity. This might be attributed to the relatively small sample of patients with XL retinoschisis.

In patients with Stickler syndrome, there was a low prevalence of comorbidities, with 22.2% having RD compared with an expected prevalence of 40 to 80% of patients developing this comorbidity. While existing literature reports a high myopia prevalence of approximately 90%, and congenital cataract in 30% of patients with Stickler syndrome, our study found notably lower rates [45]. These discrepancies may be attributed to the limited number of Stickler syndrome cases included in this study, thus highlighting the need for more collaborative studies with larger samples to have more robust conclusions.

Within the group of chorioretinal disorders, Zhou et al. reported a prevalence of cataract of 17.1% [46]. When compared to the present study, there is a 10% difference. However, this may not be clinically significant, as the findings by Zhou et al. were based on self-reported data, whereas cataract diagnosis in the present study was confirmed by ophthalmologists.

Among patients with BCD, this study identified ERM in almost a third of patients. In contrast, Dai et al. did not report any cases of ERM; rather, their study documented macular holes and even RD, none of which were observed in our cohort [46]. These differences highlight the importance of collaborative efforts among researchers to design larger, multicenter studies aimed at improving our understanding of these rare retinal disorders.

Newman et al. reported that some patients with HON may develop cataract. However, our study reported that these conditions are relatively comorbidity-free [16].

Relative to the “Other” group of this study, foveal hypoplasia had a rate of 23% of ASD, which can be corroborated by the fact that this comorbidity has been related to multiple PAX-6 disorders [47]. Jiang et al. found, in a population of 228 patients with PAX6 variants, that 76.6% had aniridia and 22.4% had iris hypoplasia. This latter study suggests a higher prevalence of ASD, which is possibly due to a difference in genetic variants in the Chinese population. Additionally, Jiang et al. reported a slightly higher prevalence of cataract [48]. Finally, in this group there was a category of Unspecified IRDs which were majorly comorbidity-free, suggesting they may be IRDs in their earlier stages, or incorrectly labeled stationary IRDs or pattern dystrophies, since these subgroups had similar rates of comorbidities.

Our study has many strengths, namely, being a collaborative study between multiple hospitals, including most tertiary care hospitals in Portugal that provide IRD patient management [49]. Also, this is the first report of IRD comorbidities in a Portuguese population, based on a national registry. Nonetheless, it also has limitations, induced by its retrospective nature, and the fact that the ocular comorbidities list is not a mandatory field in the IRD-PT registry. Thus, these may be underestimated in the current study. Also, the age at the diagnosis of each comorbidity is not available and would be beneficial to calculate an age-standardized prevalence. Additionally, potential inaccuracies in data collection due to reliance on existing records may affect the reliability of the findings. The study’s sample size for certain IRD subgroups is limited, potentially reducing statistical power and generalizability. Furthermore, the lack of comprehensive clinical testing for all patients may have led to missed diagnoses, and the absence of detailed environmental or genetic data restricts a deeper understanding of comorbidity risk factors.

## 5. Conclusions

In conclusion, this study underscores the critical role of patient registries, such as the IRD-PT, in elucidating the phenotypic landscape of IRDs. Our analysis of ocular comorbidities refined previous estimates for several IRDs, notably confirming expected rates of cataract in RP and Usher syndrome and highlighting the prevalence of CNV in PXE. While discrepancies in conditions like Stickler syndrome and X-linked retinoschisis suggest a need for further investigation, potentially reflecting sample size limitations or population-specific factors, this work collectively advances our understanding of these complex conditions. By providing a more nuanced characterization of IRD comorbidities, this study not only informs clinical management but also emphasizes the continued necessity of robust, collaborative registries to drive future research, facilitate the development of novel therapeutics, and ultimately improve patient outcomes in this evolving field.

## Figures and Tables

**Table 1:** Summary of the most frequent ocular comorbidities in IRDs.

Disease Group	Associated Comorbidities	References
Isolated progressive IRDs	Cataract, choroidal neovascularization, and refractive error	[5,10]
Isolated stationary IRDs	Foveal hypoplasia, strabismus, and myopia	[11,12]
Syndromic IRDs	Cataract, strabismus, and ophthalmoplegia	[13]
Inner retina and/or vitreoretinal dystrophies	Cataract and retinal detachment	[14]
Chorioretinal dystrophies	Choroidal neovascularization	[10]
Hereditary optic neuropathies	Cataract, ptosis, and ophthalmoplegia	[15]

**Table 2:** Clinical and demographic data of the patients included.

Characteristics	Results
Female, *n* (%)	751 (49.1%)
Male, *n* (%)	780 (50.9%)
Age, mean ± SD * (years)	45.8 ± 19.3
Age at diagnosis, mean ± SD * (years)	39.4 ± 19.5
Family history, *n* (%)	787 (52.9%)
Families, *n*	1254
Consanguinity, *n* (%)	285 (19.4%)
Number of follow-ups, mean ± SD *	4.8 ± 5.6
Visual acuity, mean ± SD * (ETDRS letters)	46.1 ± 30.1

* SD: Standard Deviation.

**Table 3:** Frequency of ocular comorbidities in the sample.

Ocular Comorbidities	Frequency, *n* (%)
None	887 (57.9%)
Cataract	324 (21.2%)
Amblyopia	96 (6.3%)
High myopia	90 (5.9%)
Glaucoma	89 (5.8%)
Pseudohole	77 (5.0%)
Cystoid macular edema	63 (4.1%)
Vitreomacular traction	58 (3.8%)
High hyperopia	55 (3.6%)
Keratoconus	50 (3.3%)
Anterior segment dysgenesis	48 (3.1%)
Strabismus	32 (2.1%)
Epiretinal membrane	31 (2.0%)
Choroidal neovascularization	26 (1.7%)
Macular hole	23 (1.5%)
Lamellar hole	20 (1.3%)
Retinal detachment	12 (0.8%)

**Table 4:** Table summarizing the different comorbidities present in patients with macular isolated progressive IRDs.

Disease	Sample Size	Associated Findings	Frequency
Stargardt disease	124 (12.3%)	None	109 (87.9%)
		Cataract	7 (5.6%)
		Amblyopia	4 (3.2%)
		Strabismus	2 (1.6%)
Best disease	28 (2.7%)	None	17 (60.7%)
		Cataract	5 (17.9%)
		CNV *	5 (17.9%)
Unspecified macular dystrophy	33 (3.3%)	None	20 (60.6%)
		Cataract	6 (18.2%)
		High myopia	2 (6.1%)
		CNV *	2 (6.1%)
		Glaucoma	2 (6.1%)

* CNV: Choroidal neovascularization.

**Table 5:** Distribution of ocular comorbidities across syndromic IRD subtypes.

Disease	Sample Size	Associated Findings	Frequency
Usher syndrome	100 (38.3%)	Cataract	43 (43.0%)
		None	40 (40.0%)
		CME *	14 (14.0%)
		ERM *	13 (13.0%)
		Strabismus	3 (3.0%)
		Glaucoma	2 (2.0%)
		Other	2 (2.0%)
		High myopia	2 (2.0%)
BBS *	37 (14.2%)	Cataract	16 (43.2%)
		None	15 (40.5%)
		Strabismus	4 (10.8%)
		ERM *	2 (5.4%)
		RD *	2 (2.7%)
PXE *	31 (11.9%)	CNV*	15 (48.4%)
		None	11 (35.5%)
		Cataract	10 (32.3%)
		Glaucoma	2 (6.5%)
Other—RP	14 (5.4%)	None	7 (50.0%)
		Cataract	3 (21.4%)
		CME *	(14.3%)
Senior–Løken syndrome	10 (3.8%)	None	4 (40.0%)
		CME *	3 (30.0%)
		Cataract	3 (30.0%)
Syndromic cone-rod/cone	7 (2.7%)	None	6 (85.7%)
C3 glomerulopathy	7 (2.7%)	None	7 (100.0%)
NBIA *	6 (2.3%)	None	4 (66.7%)
		Cataract	2 (33.3%)
Knobloch syndrome	6 (2.3%)	High myopia	4 (66.7%)
		RD *	2 (33.3%)
		Strabismus	2 (33.3%)
PHARC	5 (1.9%)	None	3 (60.0%)
Alport syndrome	5 (1.9%)	None	3 (60.0%)
		Other	2 (40.0%)
MELAS *	5 (1.9%)	None	4 (80.0%)

* BBS: Bardet–Biedl Syndrome; CNV: choroidal neovascularization; ERM: epiretinal membrane; MELAS: mitochondrial encephalomyopathy, lactic acidosis, and stroke-like episodes; NBIA: Hallervorden–Spatz syndrome; PXE: pseudoxanthoma elasticum; RD: retinal detachment.

## Data Availability

The data presented in this study are available on request from the corresponding author upon reasonable request due to sensitivity.

## Supplementary Materials

The following supporting information can be downloaded at: https://www.mdpi.com/article/10.3390/genes16070743/s1, Figure S1: Bar graph detailing the different comorbidities present in the pattern dystrophies group; Figure S2: Bar graph displaying the different comorbidities present in the isolated stationary IRDs group; Figure S3: Bar graph outlining the different comorbidities present in the inner retina and/or vitreoretinal IRDs group; Figure S4: Bar graph summarizing the different comorbidities present in chorioretinal disorders group; Figure S5: Bar graph detailing the different comorbidities present in Hereditary Optic Neuropathy group; Figure S6: Bar graph detailing the different comorbidities present in Other IRDs group.

## Abbreviations

The following abbreviations are used in this manuscript:

AD	Autosomal Dominant
AR	Autosomal Recessive
ASD	Anterior Segment Dysgenesis
BBS	Bardet–Biedl Syndrome
BD	Best Disease
CNV	Choroidal Neovascularization
CME	Cystoid Macular Edema
CSNB	Congenital Stationary Night Blindness
ERM	Epiretinal Membrane
ICD	International Classification of Diseases
IRD	Inherited Retinal Dystrophy
PXE	Pseudoxanthoma Elasticum
RD	Retinal Detachment
RP	Retinitis Pigmentosa
STGD	Stargardt Disease
ULS	Unidade Local de Saúde
XL	X-Linked
