# Supplementary material for: Exploring Concomitant Ophthalmic Comorbidities in Portuguese Patients with Inherited Retinal Diseases: A Comprehensive Clinical Study
Source: Genes (Basel). 2025 Jun 26;16(7):743. doi: 10.3390/genes16070743 (PMC12295071; doi:10.3390/genes16070743)
Supplement: Supplementary file 1 [file genes-16-00743-s001.zip › genes-3685461-supplementary.pdf]

Supplementary File #1

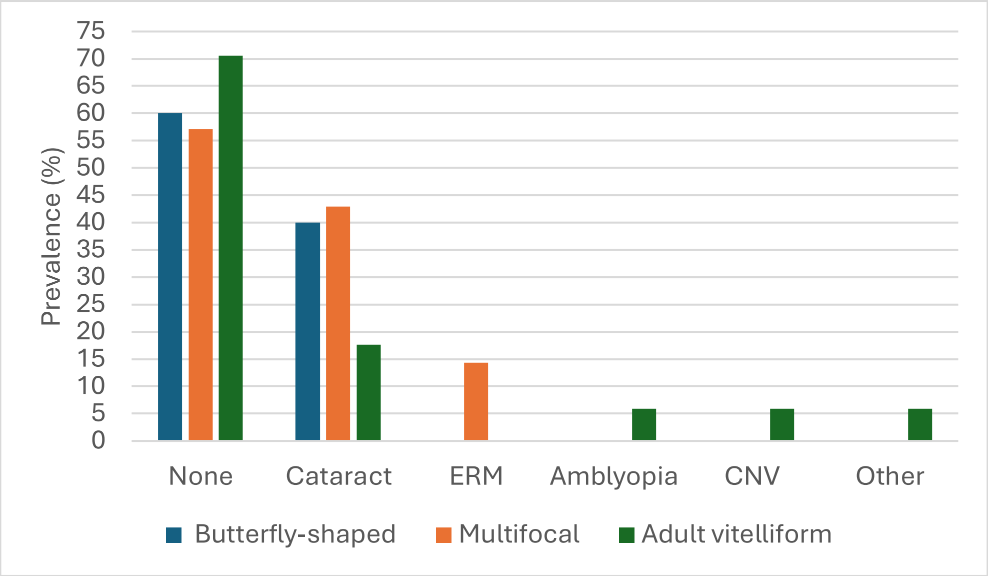

Figure S1. Bar graph detailing the different comorbidities present in the pattern dystrophies group. CME: Cystoid Macular Edema; CNV: Choroidal Neovascularization; ERM: Epiretinal Membrane

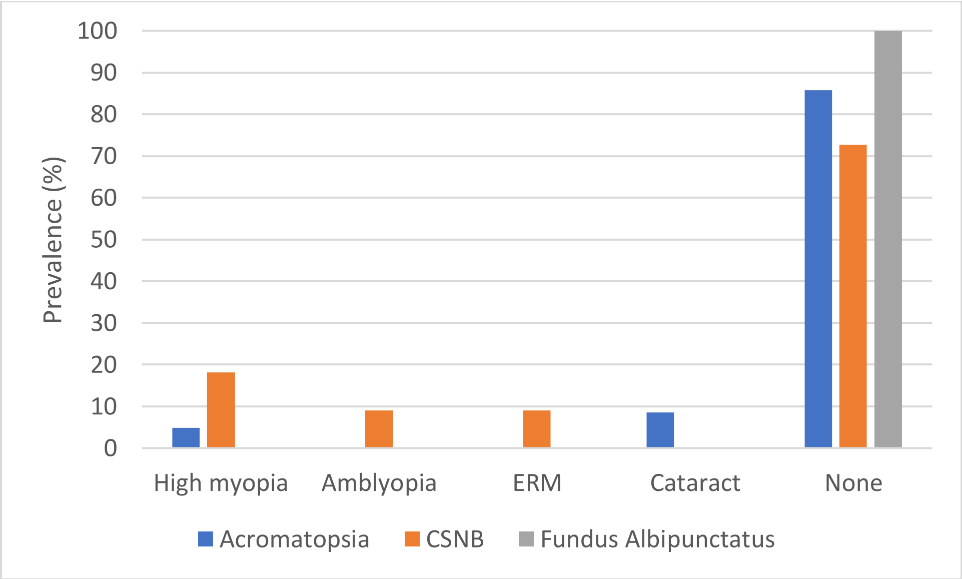

Figure S2. Bar graph displaying the different comorbidities present in the isolated stationary IRDs group. CSNB: Congenital Stationary Night Blindness; ERM: Epiretinal Membrane

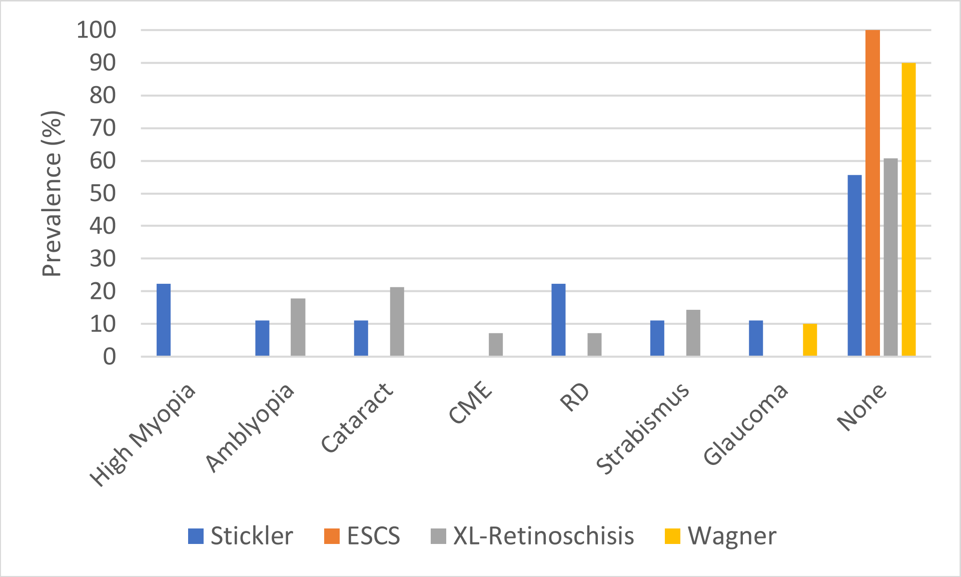

Figure S3. Bar graph outlining the different comorbidities present in the inner retina and/or vitreoretinal IRDs group. ESCS: Goldmann-Favre Syndrome; CME: Cystoid Macular Edema; RD: Retinal Detachment.

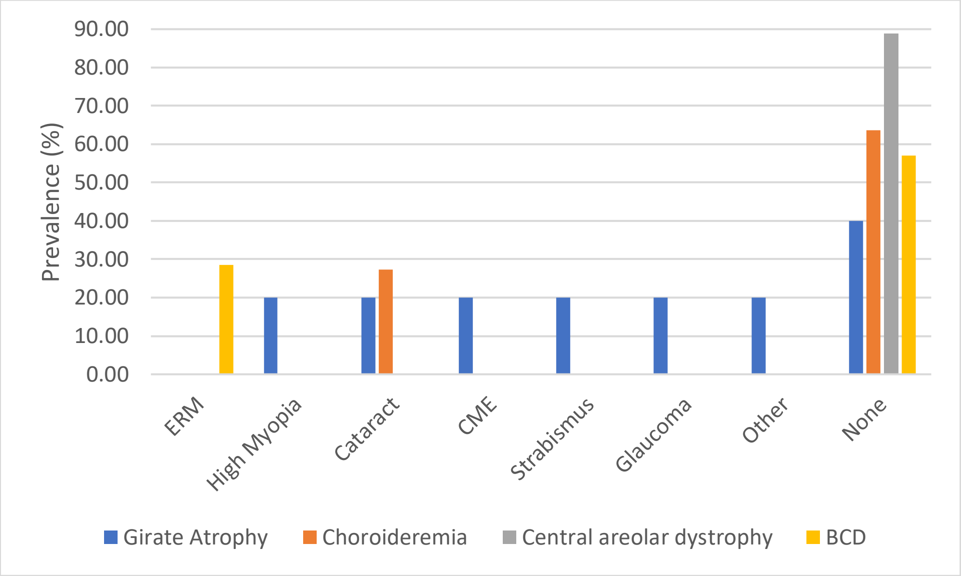

Figure S4. Bar graph summarizing the different comorbidities present in chorioretinal disorders group. BCD: Bietti Crystalline Dystrophy; CME: Cystoid Macular Edema; ERM: Epiretinal Membrane; RD: Retinal Detachment

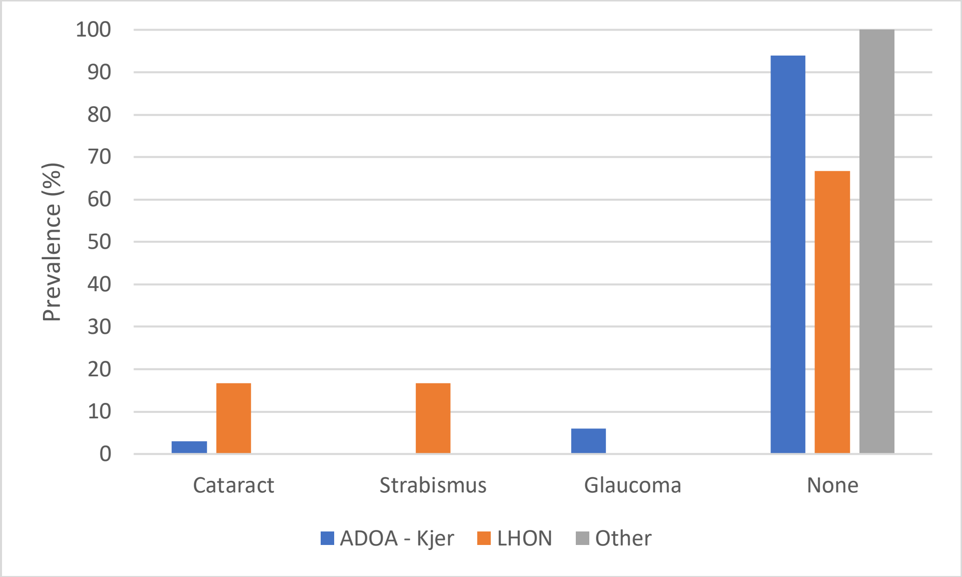

Figure S5. Bar graph detailing the different comorbidities present in Hereditary Optic Neuropathy group. ADOA – Kjer: Autosomal Dominant Optic Atrophy; LHON: Leber Hereditary Optic Neuropathy.

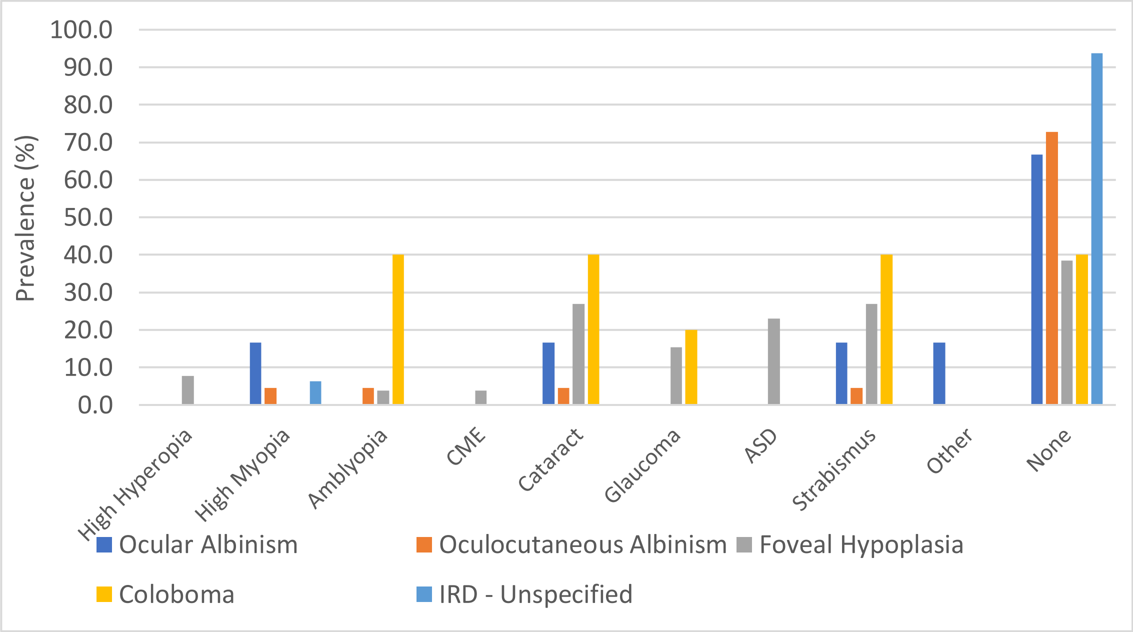

Figure S6. Bar graph detailing the different comorbidities present in Other IRDs group. CME: Cystoid Macular Edema; ASD: Anterior Segment Dysgenesis
